# Acute viral myositis – particular aspects in the epidemiology of respiratory virus diseases in 2013

**DOI:** 10.1186/1471-2334-13-S1-P90

**Published:** 2013-12-16

**Authors:** Andreea Bodea-Alucăi, Anca Georgescu, Carmen Chiriac, Brînduşa Ţilea, Cristina Gîrbovan, Nina Șincu

**Affiliations:** 1 Clinic of Infectious Diseases I, Tg-Mureş, Romania; 2 University of Medicine and Pharmacy Tg-Mureş, Romania

## Background

We aimed to highlight the incidence, clinical course and therapeutic aspects of acute viral myositis in the epidemiological context of respiratory virus diseases in 2013.

## Methods

We performed a retrospective study on the clinical, biochemical, therapeutic and prognostic of patients diagnosed with viral myositis in the Clinic of Infectious Diseases I Târgu-Mures between 01 January 2013 – 30 April 2013.

## Results

There were 578 clinically diagnosed cases of respiratory virus spread by age: 0-3 years: 40 cases (6.92%); 4-7 years: 81 cases (14.01%); 8-14 years old: 107 cases (18.51%); 15-20 years 38 patients (6.57%); over 20 years in 312 cases (53.97%); acute viral myositis was observed in a total of 22 patients (3.80%). Calendar distribution was as follows: in March, 14 cases (63.63%) compared to 6 cases (27.27%) in February, 1 case (4.54%) in January and 1 case (4.54%) in April 2013. The area of origin was urban in 18 cases (81.81%) and rural in 4 cases (18.19%). Mild clinical forms were common in 20 cases (90.9%), severe forms were determined by the prolonged evolution of the disease and association of neurological complications (polyradiculoneuritis) in 2 cases (9.1%). Positive diagnosis was clinical (myalgia, functional impotence of the lower limb) and laboratory confirmed (muscle enzymes). Evolution was favorable in all cases under treatment: corticosteroids (hydrocortisone hemisuccinate, dexamethasone medrol) and neurotrophic. Two patients received virological diagnosis, but in one of them we isolated influenza type B; this viral strain was isolated in most cases investigated virologically and confirmed with influenza viruses in patients with respiratory virus.

## Conclusion

Acute viral myositis was a complication of respiratory virus diseases of winter-spring season 2013, net prevailed at preschool age groups. The severity of the disease was low or moderate in the majority of cases, severe forms being determined by neurological damage. Therapeutic response to corticosteroid therapy was favorable.

